# Axis-Guided Vessel Segmentation Using a Self-Constructing Cascade-AdaBoost-SVM Classifier

**DOI:** 10.1155/2018/3636180

**Published:** 2018-03-18

**Authors:** Xin Hu, Yuanzhi Cheng, Deqiong Ding, Dianhui Chu

**Affiliations:** ^1^School of Computer Science and Technology, Harbin Institute of Technology at Weihai, Weihai 264209, China; ^2^School of Computer Science and Technology, Harbin Institute of Technology, Harbin 150001, China; ^3^Department of Mathematics, Harbin Institute of Technology at Weihai, Weihai 264209, China

## Abstract

One major limiting factor that prevents the accurate delineation of vessel boundaries has been the presence of blurred boundaries and vessel-like structures. Overcoming this limitation is exactly what we are concerned about in this paper. We describe a very different segmentation method based on a cascade-AdaBoost-SVM classifier. This classifier works with a vessel axis + cross-section model, which constrains the classifier around the vessel. This has the potential to be both physiologically accurate and computationally effective. To further increase the segmentation accuracy, we organize the AdaBoost classifiers and the Support Vector Machine (SVM) classifiers in a cascade way. And we substitute the AdaBoost classifier with the SVM classifier under special circumstances to overcome the overfitting issue of the AdaBoost classifier. The performance of our method is evaluated on synthetic complex-structured datasets, where we obtain high overlap ratios, around 91%. We also validate the proposed method on one challenging case, segmentation of carotid arteries over real clinical datasets. The performance of our method is promising, since our method yields better results than two state-of-the-art methods on both synthetic datasets and real clinical datasets.

## 1. Introduction

Automatic vessel segmentation in three-dimensional (3D) medical computed tomography (CT) images plays an important role in study of anatomical structure [1], in clinical diagnosis during quantification of vascular disease (tortuosity, stenosis, and calcification) [2], in vascular surgery planning [3], and in patient-specific flow simulations [1]. Vessel segmentation can help clinical workers to establish the patients' response to treatment and to determine the stage of diseases. Such applications require a competent segmentation techniques, which result in accurate segmentations not only with normal vessels but also with the presence of pathologies.

Previous work on vessel segmentation can be roughly classified into three categories. They are (a) feature-based segmentation approaches [4, 5]; (b) tracking-based segmentation approaches [6–8]; and (c) model-based approaches [9–12]. Within the previous studies, the feature-based approaches have be proven to be efficient in detecting vessels at different scales. These approaches assume that vessels have identifiable curvilinear structures [5, 13–18]. Targeting curvilinear structures, they have several common detection procedures: firstly, the eigenvalues of the Hessian matrix at each voxel are calculated at multiple scales, by convolving with 3D Gaussian filters. Then, a response function is constructed by using these eigenvalues, which can determine the shape of the local structures at a certain scale. Since vessel has curvilinear structure, we can recognize it from several nonvessel structures (planar structure, blob, noise, or no structure). The response consisting of eigenvalues can represent the local structure when it comes to its maximum over different scales. Targeting linear structure, the local maxima of the response can be used to extract vessels [5, 16]. Although these procedures output responses instead of a direct vessel segmentation, they have further advantages in structure analysis by combining Skeleton-based methods [19, 20], which resolve subsequent two-dimensional (2D) slices of vessels using tubular shape priors for ridge detection. Recently, another tracking methodology was proposed by Tyrrell et al. [21], combining 3D cylindroidal superellipsoids and local regional statistics to extract topological information from microvasculature networks. These methods are shown to be robust against noise. However, the parametrical shape priors they expect are too exclusive, which will not work with complex vessel boundaries. Another series of research works use statistical mixture model coupled with expectation-maximization algorithm including [22–24]. Since these algorithms are histogram-based, they ask for an accurate parametrical estimation or nonparametric modeling which involves density functions. They all rely on the second-order derivative information (principal curvatures of image intensities). As a result, they may suffer from sensitivity due to local deformations (e.g., blurred boundary and stenoses).

Although various sophisticated vascular segmentation algorithms have been developed in the past decade, they are still facing several challenges, such as distinguishing vessels from nonvessels with the obstruction caused by conglutination tissues, segmenting different sizes of vessels especially diseased vessels with the presence of pathologies (such as severe stenoses). These challenges can result in false detections and missed detections. Several good reviews have been published in [1–3].

Addressing the challenges proposed, we combine the vessel-specific features and embed redundancy in the feature set deliberately, to cover the widest possible spectrum of situations. We argue that the shape complexity of vessel (e.g., blurred boundary and diseased vessels) cannot be captured by a single feature. However, building an optimal feature set with strong a priori knowledge and discriminative ability involves heuristically choosing features and parameters. Thus, we propose a novel cascade-AdaBoost-SVM approach to build a feature set automatically. The AdaBoost classifier is a linear combination of multiple weak classifiers [25], in which each weak classifier only focuses and classifies one of the input features. By adding new weak classifiers in the training process, the accuracy of the AdaBoost classifier can be gradually increased. Therefore, the optimal feature set can be selected automatically along with the weak classifiers. Moreover, organizing the AdaBoost classifiers in a cascade way can help the AdaBoost classifier focus on identifying vessels from vessel-like structures. And combining the SVM classifier can make the cascade-AdaBoost classifier avoid the overfitting issue.

This paper is organized as follows.  Section 2 introduces the cascade-AdaBoost-SVM classifier. In Section 2, Section 2.1 details the feature set adopted. This cascade-AdaBoost-SVM classifier works on cross-sections based on a vessel axis + cross-section model, which is given in Section 2.2. We then introduce the training samples for the proposed algorithm in Section 2.3. The details of self-constructing cascade-AdaBoost-SVM classifier are presented in Section 2.4. The dataset, the evaluation methods, and the experimental results are shown in Section 3. Finally, we discuss the results and draw conclusion in Section 4.

## 2. Method

This paper describes a vessel axis + cross-section model for carotid artery, which utilizes a self-constructing classification algorithm. First, we extract vessel axis from a gray-scale 3D angiogram, by using our previous method [9]. Along this extracted vessel axis, the vessel axis + cross-section model is constructed on the cross-section. Subsequently, a feature set for the self-constructing classification algorithm is presented, followed by a review of the AdaBoost machine learning algorithm. Finally, we introduce the self-constructing cascade-AdaBoost-SVM classifier.

### 2.1. Feature Set for Classification Algorithm

On CT images, the vessel intensity may vary within a relatively wide range, due to blood flow rate and vessel dimensions. Vessels with various diameters can be regarded as 3D line structures. Therefore, the feature set was selected to enable detection of various line structures and sizes for vessels. The eigenvalues of the Hessian matrix are used to calculate the gradient based shape features. A multiscale approach is used to improve the detection of various size line structures.

Let $H=\nabla^{2}I(\vec{x})$ be the Hessian matrix of a 3D image $I(\vec{x})$ about an arbitrary point $\vec{x}=[x\;y\;z]$, and the eigenvalue vector $\lambda (\vec{x})=[\lambda_{1}(\vec{x})\;\lambda_{2}(\vec{x})\;\lambda_{3}(\vec{x})]$ with $\lambda_{1}(\vec{x})>\lambda_{2}(\vec{x})>\lambda_{3}(\vec{x})$ be the eigenvalues of *H* with corresponding eigenvectors given by $e_{1}(\vec{x})$, $e_{2}(\vec{x})$, and $e_{3}(\vec{x})$, respectively. Using the matrix of the eigenvectors, we have

(1) ET∇2Ix→E=e3x→e2x→e1x→T∇2Ix→e3x→e2x→e1x→=λ3x→000λ2x→000λ1x→.

Equation (1) describes the second-order structure of local intensity variations around each point of $I(\vec{x})$ extracted by the matrix *H*, and the local second-order features of $I(\vec{x})$ obtained by the eigenvalues ($\lambda_{1}(\vec{x})$, $\lambda_{2}(\vec{x})$, and $\lambda_{3}(\vec{x})$).

The profile of a line structure on the cross-section can be supposed to have a 2D Gaussian shape:

(2) $$\begin{array}{c} L\left(\;{\vec{x}}^{\prime }\;\right)=\exp\left(\;-\frac{x^{\prime 2}+y^{\prime 2}}{2\sigma_{r}^{2}}\;\right), \end{array}$$

where ${\vec{x}}^{\prime }=[x^{\prime },y^{\prime }]$ is an arbitrary point on the cross-section centered at an axis point $\vec{c}=[c_{x}\;c_{y}\;c_{z}]$; the standard deviation *σ*_*r*_ is related to the scale of line structures; [*x*′ *y*′ 0]^*T*^ = *R*[*x* − *c*_*x*_ *y* − *c*_*y*_ *z* − *c*_*z*_]^*T*^, where [*x*, *y*, *z*] is the coordinate of point ${\vec{x}}^{\prime }$ in 3D; *R* is a 3 × 3 rotation matrix. This cross-section at the axis point $\vec{c}$ can be determined by using the corresponding eigenvectors $e_{1}(\vec{c})$, $e_{2}(\vec{c})$, and $e_{3}(\vec{c})$. The eigenvectors point out singular directions: $e_{1}(\vec{c})$ indicates the direction along the vessel, which is normal to the cross-section plane, while $e_{2}(\vec{c})$ and $e_{3}(\vec{c})$ form a base for the cross-section plane [14].

The 2D Gaussian shape (see (2)) is a mathematical line model for the vessels, while the Hessian matrix (see (1)) is used to extract local shape features. Combining these two equations (see (1) and (2)), researchers derive measures of similarity between vessels and the line structure [5, 14, 26–28]. The similarity measures can be considered as vessel-specific features. The scales used for this Gaussian shape in this study, *σ*_*r*_ = 0.6 × 2^(*i*−1)/2^, *i* = 1,2,…, 6, correspond to the sizes of the line structures (vessels), which are 0.7, 1.0, 1.6, 2.4 3.5, and 6.0 mm. These scales allow both large and small line structures to be detected. However, at small scales, the boundary of large line structures may not be accurately captured due to noise and small inhomogeneities in the structure. On the other hand, at large scales, the shape of small line structures may be distorted as neighboring structures. This is the reason we combine multiple vessel-specific features, which embed shape and scale redundancy, to cover the widest possible spectrum of situations. In the following, we describe the multiple vessel-specific features in the feature set.

(*1) Sato Feature (f*_1_*) [5]*. This measure is suitable for images, where vessels are with bright tubular structures in a dark environment. For each scale, *σ*_*r*_, the Sato vesselness measure, *f*_1_, is given by

(3) f1λ1,λ2=λ2·exp⁡−λ122α1λ22λ1≤0,  λ2≠0λ2·exp⁡−λ122α2λ22λ1>0,  λ2≠00λ2=0,

where *λ*_1_ and *λ*_2_ are the eigenvalues of *H* defined in (1). *α*_1_ and *α*_2_ are two preset parameters, *α*_1_ < *α*_2_. *α*_1_ and *α*_2_ were fixed to 0.5 and 2, respectively.

(*2) Frangi Feature f*_2_* [14]*. This measure is a nonlinear combination of the eigenvalues of the Hessian matrix that promotes the enhancement of line structures, while noise and non-line-like structures are smoothed out. At a single scale, *σ*_*r*_, the Frangi vessel-dedicated feature, *f*_2_, is given as follows:

(4) $$\begin{array}{c} f_{2}\left(\;\lambda_{1},\lambda_{2},\lambda_{3}\;\right)=\left\{\begin{array}{cc} 0 & \lambda_{2}>0\text{ or }\;\lambda_{3}>0\\ \left[1-\exp\left(-\frac{R_{A}^{2}}{2\alpha^{2}}\right)\right]\cdot \exp\left(-\frac{R_{B}^{2}}{2\beta^{2}}\right)\cdot \left[1-\exp\left(-\frac{S^{2}}{2\gamma^{2}}\right)\right] & \text{otherwise,} \end{array}\right. \end{array}$$

where *R*_*A*_ = |*λ*_2_|/|*λ*_3_| (subject to *α*) discriminates plate-like structures from line-like structures, $R_{B}=\left|\lambda_{1}\right|/\sqrt{\left|\lambda_{2}\lambda_{3}\right|}$ (subject to *β*) discriminates blob-like structures from line-like structures, and $S=\sqrt{\lambda_{1}^{2}+\lambda_{2}^{2}+\lambda_{3}^{2}}$ (subject to *γ*) eliminates background noise. We set *α* = 0.5, *β* = 0.5 in this study. And *γ* is equal to half of the maximum Frobenius norm of the Hessian over all Frobenius norms computed on the whole image.

(*3) Shikata Feature f*_3_* [26]*. This model assumes that the vessel has a cylindrical shape with 2D Gaussian intensity distribution on its cross-section. This assumption meets our application, which is a vessel axis + cross-section model-based approach. Moreover, this model is able to enhance the small vessels. This Shikata tubular feature, *f*_3_, is defined as follows:

(5) $$\begin{array}{c} f_{3}\left(\;\vec{x},\lambda_{2}\;\right)=\sigma_{r}^{2}\cdot \frac{\lambda_{2}}{I\left(\vec{x}\right)}, \end{array}$$

where $I(\vec{x})$ is the intensity at point $\vec{x}$ defined in (1).

(*4) Li Feature f*_4_* [27]*. This model is a selective enhancement, which uses curvature analysis to identify structures with specific shapes. Due to this unique selective characteristic, Li feature can be potentially useful to distinguish tubular objects from other structures. This Li tube-specific feature, *f*_4_, is given as follows:

(6) $$\begin{array}{c} f_{4}\left(\;\lambda_{1},\lambda_{2},\lambda_{3}\;\right)=\left\{\begin{array}{cc} \frac{\left|\lambda_{2}\right|\left(\left|\lambda_{2}\right|-\left|\lambda_{3}\right|\right)}{\left|\lambda_{1}\right|} & \lambda_{1}<0,\;\;\lambda_{2}<0\\ 0 & \text{otherwise.} \end{array}\right. \end{array}$$

*(5) Manniesing Feature f*_5_ *[28]*. Manniesing et al. improve Frangi's method [14] by applying a nonlinear anisotropic Hessian-based diffusion along the local line directions. Due to the steering of diffusion, this model has strong isotropic diffusion to reduce background noise. This diffusion feature, *f*_5_, is defined as follows:

(7) $$\begin{array}{c} f_{5}\left(\;\lambda_{1},\lambda_{2},\lambda_{3}\;\right)=\left\{\begin{array}{cc} 0 & \lambda_{2}\geq 0\text{ or }\lambda_{3}\geq 0\\ \left[1-\exp\left(-\frac{R_{A}^{2}}{2\alpha^{2}}\right)\right]\exp\left(-\frac{R_{B}^{2}}{2\beta^{2}}\right)\left[1-\exp\left(-\frac{S^{2}}{2\gamma^{2}}\right)\right]\exp\left(-\frac{2c^{2}}{\left|\lambda_{2}\right|\lambda_{3}^{2}}\right) & \text{otherwise,} \end{array}\right. \end{array}$$

where *R*_*A*_, *R*_*B*_, *S*, *α*, *β*, and *γ* are defined in (4); *c* is the smoothness constant of the vesselness function. By following Manniesing et al. [28], the smoothed vessel filter has parameters: *α* = *β* = 0.5, *γ* = 5, and *c* = 10^−6^.

### 2.2. Vessel Axis + Cross-Section Model

In our previous method [9], vessel axes are detected in three stages. Firstly, vessel regions are enhanced and extracted by applying multiscale filtering method based on Hessian matrix. Since the extracted vessel regions are not very accurate, these regions are used as masks for vessel axis points detection subsequently. Finally, tracking and connecting the axis points at multiple scales reconstruct continuous axes and their branching structures.

**Figure 1:**
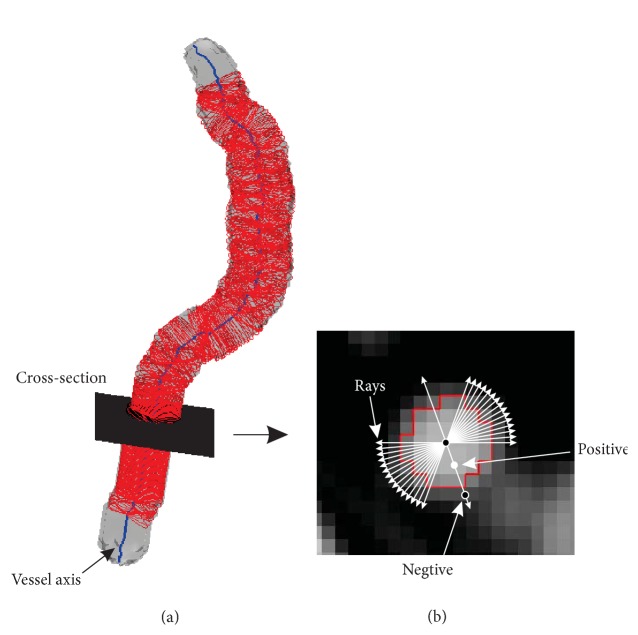
Vessel axis + cross-section model. (a) 2D vessel cross-sectional plane orthogonal to the vessel axis. (b) Rays for training sample collection and vessel detection. Rays are spaced every 5° from the axis point on the cross-section. We just show part of them for example. The red circle on the cross-section is the manual marked boundary. The points (white point) inside the boundary along the ray are collected as positive training samples (vessel class), while the points (black point) outside the boundary are marked as negative training samples (nonvessel class).

Figure 1 shows the cross-section model on a cross-section orthogonal to the vessel axis. As we discussed previously, the cross-sections can be determined by using the eigenvectors of axis points. Assume that the profile on this cross-section is not perfectly round; 72 rays are spaced every 5° from the axis point on the cross-section. Along these rays, we collect training samples or detect vessel boundaries.

### 2.3. Manual Segmentations as Training Samples

We collected 30 CT datasets acquired by our collaborators (the 2nd Affiliated Hospital of Harbin Medical University). These datasets were selected from 95 patients with known carotid stenosis. These 95 patients, 63 male and 32 female participants with mean age 68 years (min 58, max 91), entered our study nonconsecutively, during the period from December 2012 to December 2016. These 30 datasets were segmented by two expert raters.

As far as we know, the ground truth segmentations of those clinical data sets do not exist, even segmented by multiple experts. Thus, we asked two expert raters to compose segmentations by following a segmentation protocol: marking boundaries in the slices of image, tracking the vessel tree and marking the boundaries of the vessels semiautomatically by using a graphical user interface (GUI) [9]. This GUI can propagate to mark the vessel boundaries in 3D image automatically, after the user marks the boundaries in some slices (not asking for continuous slices). In the propagating process, user can take a view in sagittal, axial, or coronal direction. The user can also check the propagating process by using the GUI functions: cine-paging through the slices, scrolling in and out of individual vessel, adjusting window setting, and zooming in and out to improve the visualization in one direction. With the marked vessel boundaries, training samples were collected along the rays on the cross-section, as shown in Figure 1. Positive training samples are collected as vessel class, and negative training samples belong to the nonvessel class.

### 2.4. Self-Constructing Cascade-AdaBoost-SVM for Vessel Detection

In this subsection, we introduce a self-constructing cascade-AdaBoost-SVM classifier, which organizes the AdaBoost classifiers in a cascade way and combines the AdaBoost classifier [25] with SVM [29]. Firstly, we introduce the cascade-AdaBoost classifier [30, 31] and then proceed with the self-constructing cascade-AdaBoost-SVM classifier.

#### 2.4.1. Cascade-AdaBoost Classifier

The AdaBoost classifier is a parallel classifier, which combines many linear weak classifiers. After the goal is given to the AdaBoost classifier, the AdaBoost training algorithm can increase the weak classifiers to achieve this goal self-adaptively. Since each weak classifier just focuses on the classification of one dimension in the given feature set, the AdaBoost classifier can focus on several key features by adding weak classifiers. After a weak classifier is added, the minimum error is employed to calculate the weight value of this weak classifier and readjust the weight values for all the training samples and then take these weight values as the input of next training iteration. By adding a new weak classifier in each training iteration, the capability of the overall AdaBoost classifier is improving. At last, the final result of the strong classifier *H*(*F*) is expressed in the following equation:

(8) $$\begin{array}{c} H\left(\;F\;\right)=\overset{T}{\underset{t=1}{\sum }}\;\beta_{t}h_{t}\left(\;f_{i}\;\right), \end{array}$$

where *F* is the feature set described in Section 2.2, *F* = {*f*_1_, *f*_2_, *f*_3_, *f*_4_, *f*_5_}; *h*_*t*_ is the weak classifier added in the *t*th training iteration; *f*_*i*_ is the key feature selected by the classifier *h*_*t*_; *β*_*t*_ represents the corresponding weight value of each weak classifier; *T* is the number of training iterations and the total number of weak classifiers after the training process.

Since we explore the discrimination of features by training weak classifiers and organize the AdaBoost classifiers in a cascade way, we ask for simple weak classifiers, with which the target of the cascade-AdaBoost classifier can be easily controlled. Thus, simple threshold classifiers are chosen as weak classifiers as follows:

(9) $$\begin{array}{c} h_{t}\left(\;f_{i}\;\right)=\left\{\begin{array}{cc} 1 & T_{\text{lower}}\leq f_{i}\leq T_{\text{upper}}\\ 0 & \text{otherwise,} \end{array}\right. \end{array}$$

where *T*_lower_ and *T*_upper_ are thresholds for the weak classifier *h*_*t*_, which can be obtained by using a semiexhaustive search technique [32].

As we focus on accurate segmentation of vessels with challenging cases (e.g., blurred boundaries and vessel-like structures), the simplest method to achieve this goal is to initiate the positive training samples and negative training samples with different weight values. Let {*f*_*i*_, *y*_*i*_} be the training samples, *f*_*i*_, *i* = 1,…, 5 is the feature set, *y*_*i*_ ∈ {1, −1} represents vessel class or nonvessel class. Suppose there are *p* positive samples and *q* negative samples in the training set, the weight values of positive samples and negative samples, *w*_*p*_ and *w*_*n*_, can be initiated as follows:

(10) wp=q·wnp·wp+q·wn=1,

where *w*_*p*_ and *w*_*n*_ are the weight values of positive samples and negative samples, respectively; *p* and *q* are the numbers of positive samples and negative samples.

By following (10), the weight values of the positive samples and negative samples would be set as 1/(*p* + 1) and 1/*q*(*p* + 1), before the training process. Then, the training loop of *T* iterations is cycled. When carrying out the *t*th training cycle, 5 weak classifiers will be trained for 5 features. Among these weak classifiers, the weak classifier with the lowest error is selected as the weak classifier *h*_*t*_ of the *t*th cycle. The feature corresponds to the weak classifier *h*_*t*_ which is also selected. The corresponding weight value *β*_*t*_ of this weak classifier *h*_*t*_ is calculated based on the training error. The weight value *w*_*j*_(*t* + 1), *j* = 1,…, *m* of each training sample is then adjusted to pass on the priority of each training sample to the (*t* + 1)th cycle; *m* is the number of all the training samples, *m* = *p* + *q*. Finally, the sum of all the *T* weak classifiers and the corresponding weight values *β*_*t*_ are calculated. The strong classifier *H*(*F*) as the sum of these *T* weak classifiers can be obtained.

Since we are exploiting a multiscale vessel detection method, the strong classifier in (8) can be rewrote as

(11) $$\begin{array}{c} H\left(\;F\;\right)=\underset{s}{\max}\overset{T}{\underset{t=1}{\sum }}\;\beta_{t}^{s}h_{t}^{s}\left(\;f_{i}^{s}\;\right), \end{array}$$

where *f*_*i*_^*s*^, *i* = 1,…, 5 are the features at scale *s*; for each scale *s*, we train *T* weak classifiers and calculate corresponding weight values *β*_*t*_^*s*^.

Although the AdaBoost classifier can increase the overall accuracy of segmentation gradually by adding new weak classifiers, it would fail with challenging cases (e.g., blurred boundaries and vessel-like structures). Since the vessels and nonvessels in the challenging cases are with similar intensity, similar shape, or both, the AdaBoost classifier cannot identify vessels from nonvessels. Addressing this challenge, we organize multiple AdaBoost classifiers in a cascade way by using cascade-AdaBoost classifier [30], which can increase the accurate detection while reducing the false positive detection. Here, we would like to specify three definitions: (1) accurate detection is the sample in vessel class and detected as vessel; (2) false positive is the sample in nonvessel class and detected as vessel; (3) true negative is the sample in nonvessel class and detected as nonvessel.

**Figure 2:**
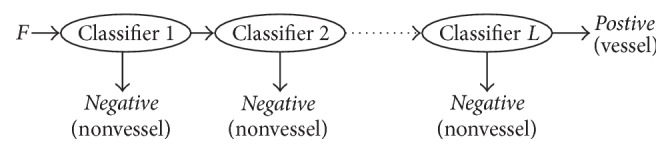
Cascade-AdaBoost architecture diagram. Each classifier is an AdaBoost classifier.

The cascade-AdaBoost classifier architecture is shown in Figure 2. If the input feature vector, *F* = {*f*_1_,…, *f*_5_}, selected by the AdaBoost classifier, is determined as a negative sample, it would be removed from the training set when passing the first AdaBoost classifier, without entering next AdaBoost classifier. As a result, the number of negative samples in training set would be decreased along with the increase in layers. In the contrary, if the input feature vector, *F*, is determined as a positive sample, this training sample could enter next layer for further classification. And this positive training sample will not stop until it reaches the last layer. Therefore, the training samples on the rear layers are similar to each other, which makes the AdaBoost classifiers on the rear layers focus on similar training samples. As a result, the cascade-AdaBoost classifier has the capability to identify vessel voxels from vessel-like voxels.

Assume that there are *L* layers in this cascade classifier, and the detection rate and false positive rate of each layer are set to *d*_*i*_ and *e*_*i*_, respectively. Then the detection rate and the false alarm rate of the whole cascade classifier can be calculated: *D* = (*d*_*i*_)^*L*^ and *E* = (*e*_*i*_)^*L*^, respectively. Here, we take 10-layer cascade classifier, for example. The detection rate *d*_*i*_ and the false positive rate *e*_*i*_ for each layer are set as 0.99 and 0.3, respectively. Then, the whole detection rate *D* and the whole false positive rate *E* can be 0.99^10^ > 0.9 and 0.3^10^ < 6*e* − 6. In order to identify vessel from vessel-like structure, the cascade-AdaBoost classifier needs to maintain high detection rate and low false positive rate on each layer.

We found that the AdaBoost classifiers on front layers could reach preset targets with less weak classifiers, but those classifiers on the rear layers fail to do so, even with more weak classifiers. In the training process, the training set removes some negative samples when passing through each layer of the cascade-AdaBoost classifier. With the increase of layers, the remaining negative samples become much less than the positive samples. This will lead to overfitting issue of the AdaBoost classifier. Here, the AdaBoost classifier is not a perfect classifier for positive and similar negative samples. To solve this problem, we propose a self-constructing cascade classifier, combining AdaBoost classifier with SVM.

#### 2.4.2. Self-Constructing Cascade-AdaBoost-SVM Classifier

At the very beginning of the training process, we set the AdaBoost classifier on each layer with the lowest acceptable detection rate, the highest acceptable false positive rate, and the maximum number of weak classifiers. When the AdaBoost classifier could not achieve the preset performance (the lowest acceptable detection rate and the highest acceptable false positive rate) under the predetermined maximum number of weak classifiers, substitute this AdaBoost classifier with SVM and train SVM with the feature set selected by the AdaBoost classifier. Before achieving the overall goals, this cascade classifier will increase layer (AdaBoost or SVM) adaptively. This is the proposed self-constructing cascade-AdaBoost-SVM, as shown in Algorithm 1.

Before presenting the cascade-AdaBoost-SVM algorithm, we would like to introduce SVM briefly. With similar training samples, linear classifiers (including AdaBoost classifier) would not work. Thus, we hope to find a hyperplane $\xi (\vec{f})$, which makes vessel class fall into the range of $\xi (\vec{f})>0$ and nonvessel class fall into the range of $\xi (\vec{f})<0$. Here, $\vec{f}$ is the feature vector selected by the AdaBoost classifier. We can classify voxels according to the sign of $\xi (\vec{f})$. The hyperplane $\xi (\vec{f})$ can be expressed as

(12) $$\begin{array}{c} \xi \left(\;\vec{f}\;\right)={\vec{w}}^{T}\cdot \vec{f}+b, \end{array}$$

where $\vec{w}$ is the normal vector of this hyperplane and $-b/\left\|\vec{w}\right\|$ is the distance from the origin perpendicular to the hyperplane. We can search for this $\vec{w}$. Then, the solution of SVM is based on minimum square error as follows:

(13) $$\begin{array}{c} E\left(\;\vec{w},b\;\right)=\min\left\{\;\frac{1}{2}{\left\|\;\vec{w}\;\right\|}^{2}-\overset{m}{\underset{i=1}{\sum }}\alpha_{i}{\left(\;y_{i}-\xi \left(\;\vec{f}\;\right)\;\right)}^{2}\;\right\}, \end{array}$$

where *α*_*i*_ > 0, *i* = 1,…, *m* are the coefficients of Lagrange. Here, we use the Gaussian function (2) as the kernel function.

During the training process of the cascade-AdaBoost-SVM classifier (Algorithm 1), we firstly set the minimum acceptable detection rate *d*, the maximum acceptable false positive rate *e*, and the maximum number of weak classifiers *n*_th_ for each layer. The overall false positive rate *E*_target_ for the cascade classifier is selected. Let **P** and **N** represent positive training sample set (vessel) and negative training sample set (nonvessel), respectively. The training process of the cascade classifier is mainly composed of two loops: (1) the internal loop is used to train classifiers, AdaBoost or SVM, for each layer. Each time in the loop, the AdaBoost classifier focuses on one feature by selecting a weak classifier. Then the present AdaBoost classifier will be reappraised to see whether it has satisfied the preset conditions (the minimum acceptable detection rate *d* and the maximum acceptable false positive rate *e*). If it has not, continue adding weak classifiers until conditions of the inner loop are satisfied. Otherwise, it determines whether the number *n*_*i*_ of weak classifiers is larger than the maximum value *n*_th_. If it is, the AdaBoost classifier will be substituted by a SVM classifier on this layer. And this SVM classifier can be trained with the features selected by the AdaBoost classifier. The training process of SVM can be completed more effectively, since it avoids the heavy burden of calculating all features as input vector. (2) The external loop controls the overall false positive rate for the present cascade-AdaBoost-SVM classifier. Since we just remove true negative training samples out of **N** on each layer and leave all the positive training samples to pass through all the layers, the cascade-AdaBoost-SVM classifier can maintain high detection rate by constraining the overall false positive rate. The parameters of the proposed cascade-AdaBoost-SVM classifier are set as follows: *d* = 0.99, *e* = 0.3, *n*_th_ = 15, and *E*_target_ = 6*e* − 6.

## 3. Results

In order to evaluate the performance of the proposed cascade-AdaBoost-SVM classifier and to illustrate the capability of the method in delineating accurate vessel boundary with diseased vessels, we tested it in one synthetic vascular experiment and one challenging clinical case, carotid artery segmentation.

The results of our work are compared with two state-of-the-art methods: a vessel tractography method [33] and a learning-based regression method [12]. Cetin et al. define a vessel tensor and combine it with a vessel centerline tracing method. This vessel tensor models the vessel as a cylinder, which is an explicitly geometrical feature. And Schaap's method learns the geometry and the appearance of the vessels from annotated data. Both of them have been proved to have the capability in identifying vessels from vessel-like structures.

### 3.1. Evaluation Metrics and Statistical Tests

In order to evaluate the proposed method and the two previous methods, quantitative analysis is employed here, which calculates the value of overlap and accuracy by comparing the segmented vessels with the manual reference segmentations. For the purpose of quantitative analysis, we borrowed two volume- and surface-based metrics from literatures: Dice Overlap Coefficient (DOC) [34] and Average symmetric Surface Distance (ASD) [35]. For DOC (ASD), the larger (smaller) the value is, the better the segmentation result is. And the DOC (ASD) is given in percent (millimeters).

To present the comparison results more clearly, the paired *t*-test was employed to assess the differences in segmentation accuracy between our method and other methods. The differences are considered to be statistically significant with a significance level set at *p* < 0.05. And the significant differences are marked with symbol *∗*.

### 3.2. Synthetic Vascular Datasets

**Figure 3:**
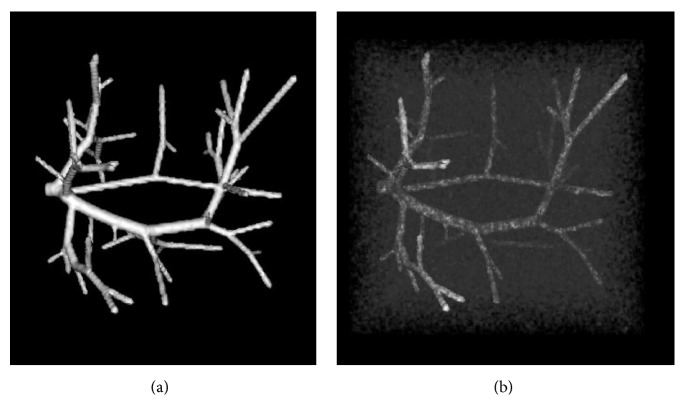
Synthetic vessel tree example from the synthetic vascular dataset. The original image (a) and blurred image with Gaussian noise (b).

The synthetic vascular datasets simulate the vascular trees and generate the corresponding ground truth segmentations, which are obtained from Hamarneh and Jassi's work [36]. Since the blurred vessel boundary is one of the main challenges for accurate vessel segmentation, we evaluate the performance of the three algorithms by adding different levels of Gaussian noise, as shown in Figure 3.

For simplicity, we used the three sample datasets available online in our synthetic validation experiments. To simulate the blurred vessel boundaries, Gaussian noise with different levels, *σ*_noise_^2^ = 20, *σ*_noise_^2^ = 40, *σ*_noise_^2^ = 60, and *σ*_noise_^2^ = 80, is added to the images to form two datasets (one for training and the other one for testing).

**Figure 4:**
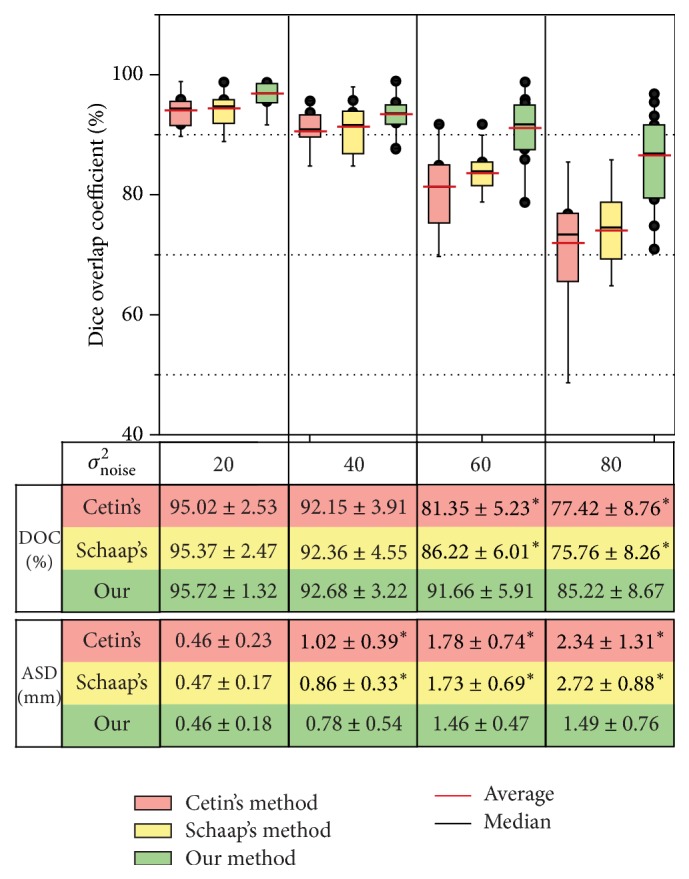
Summary of comparison evaluation on the 12 synthetic vascular volumes. Dice overlap coefficients (DOC) using the three methods are plotted. The averages of DOC and average ASD and statistical significance are shown in a table. In this table, *∗* indicates the statistically significant differences between our method and the other two at a significance level of 0.05.

The three methods were tested on the 12 synthetic vascular volumes, which are generated from the three synthetic vascular datasets by adding four different levels of Gaussian noise. The comparison results are summarized in Figure 4. We can observe that Cetin's method yields a high average DOC of 95.02 ± 2.53%, 92.15 ± 3.91%, 81.35 ± 5.23%, and 77.42 ± 8.76% for the four levels of noise, respectively. Schaap's method gives a similar result of 95.37 ± 2.47%, 92.36 ± 4.55%, 86.22 ± 6.01%, and 75.76 ± 8.26%. Our method achieves an average DOC of 95.72 ± 1.32%, 92.68 ± 3.22%, 91.66 ± 5.91%, and 85.22 ± 8.67%.

We then present the comparison results by using ASD. An average ASD of 0.46 ± 0.23 mm, 1.02 ± 0.39 mm, 1.78 ± 0.74 mm, and 2.34 ± 1.31 mm is obtained by the Cetin's method. These values are changed to 0.47 ± 0.17 mm, 0.86 ± 0.33 mm, 1.73 ± 0.69 mm, and 2.72 ± 0.88 mm by the Schaap's method, and the values are improved to 0.46 ± 0.18 mm, 0.78 ± 0.54 mm, 1.46 ± 0.47 mm, and 1.49 ± 0.76 mm by our method. These results shown in Figure 4 demonstrate that the proposed method is more resistant to Gaussian noise, compared to the other two methods, and the differences between our method and the other two are statistically significant (*p* < 0.05) with high levels of noise.

**Figure 5:**
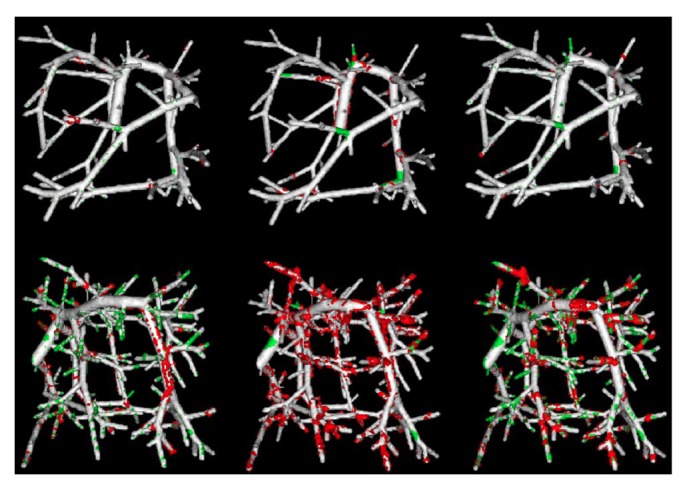
Comparison results on the randomly selected synthetic vascular data with the presence of two levels of Gaussian noises *σ*_noise_^2^ = 20 (top) and *σ*_noise_^2^ = 80 (bottom). First column shows our segmentations, the second column shows Cetin's segmentations, and the third column shows Schaap's segmentations. The overlapping points between each of the computational methods and the corresponding ground truth are shown in gray; the points that belong to the ground truth but not extracted by the computational methods are shown in green, and the points that are detected by the computational methods but do not belong to the ground truth are shown in red.

Figure 5 shows 3D views of segmentation results on one synthetic vascular dataset with the Gaussian noise of *σ*_noise_^2^ = 20 and *σ*_noise_^2^ = 80. It can be observed that the segmentations obtained by our method are more accurate than those of Cetin's and Schaap's methods.

### 3.3. Carotid Artery Datasets

**Figure 6:**
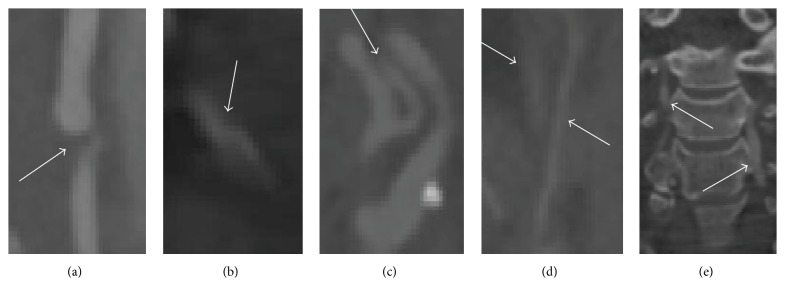
Examples demonstrating several challenges of vessel extraction in carotid artery images. Arrows indicate the vessels with different challenges: (a) stenosis, (b) kissing and low contrast vessels, (c) kissing vessels, (d) low contrast vessels, and (e) touching bone.

Carotid artery stenosis is a narrowing or constriction of the inner lumen of the carotid artery, which can increase the risk for ischemic stroke. In order to reduce the overall stroke risk, medical intervention (e.g., carotid endarterectomy and carotid stenting) asks for accurate segmentation of carotid artery, which is really valuable for surgery planning. However, the accurate segmentation of carotid artery is challenging, due to the present of stenosis, kissing vessels, low contrast vessels, and touching bone, as shown in Figure 6.

Addressing the proposed challenges (Figure 6), we conducted our experiments on 30 carotid CT images (120 kV, 40 mAs). These images have a slice thickness of 1 mm and in-plane voxel size ranging from 0.72 to 0.78 mm. We selected 10 images randomly for training and conducted the comparison experiment on the remaining images. Note that these 30 images are marked by two raters manually.

**Figure 7:**
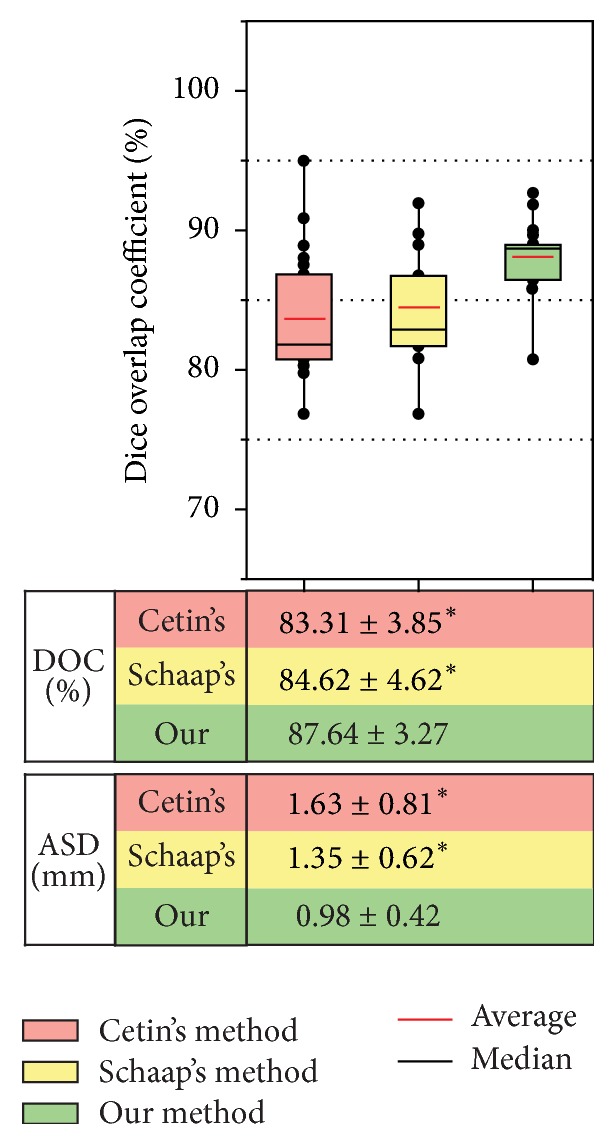
Summary of accuracy evaluation on 30 carotid artery datasets for the three methods. DOCs using Cetin's, Schaap's, and our methods are plotted. Average DOC and ASD values and statistical significance are also shown just below the box plot in a table. In the table, symbol *∗* indicates the statistically significant differences between our method and the other two.

The comparison results of the three methods are summarized in Figure 7. As we can observe, on average of 20 carotid artery datasets, DOCs are 83.31  ±  3.85%, 84.62  ±  4.62%, and 87.64  ±  3.27% for the Cetin's and Schaap's methods and our methods, respectively, while ASDs are 1.63  ±  0.81 mm, 1.35  ±  0.62 mm, and 0.98  ±  0.42 mm. The comparison results show that our method achieves the best segmentation accuracy. Besides, the differences in performance between our method and the other two are statistically significant (*p* < 0.05).

**Figure 8:**
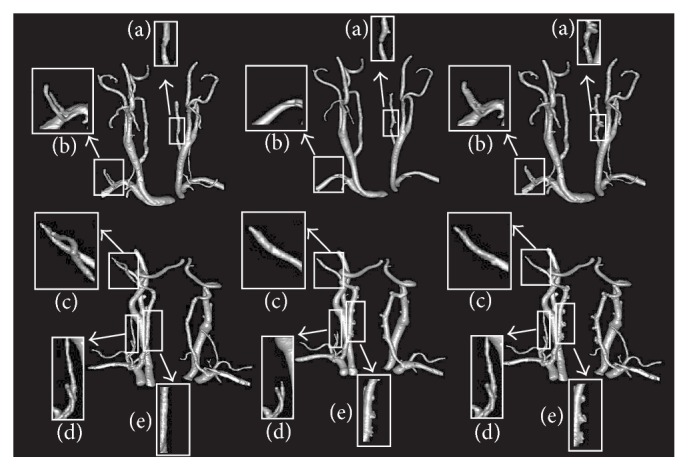
Results of two challenging carotid artery cases for the three methods. The two rows illustrate the two different carotid arteries. First column shows our segmentations, the second column shows the Cetin's segmentations, and the third column shows Schaap's segmentations. White rectangles indicate the differences of the three methods in detecting vessels with the challenges shown in Figure 6: (a) stenosis, (b) kissing and low contrast vessels, (c) kissing vessels, (d) low contrast vessels, and (e) touching bone.

As far as we know, the vessels in the presence of pathologies, shown in Figure 6, need to be located and segmented accurately before surgery. Here, we take two carotid arteries with the same challenges, for example, and show the comparison results in 3D (Figure 8). The white rectangles in Figure 8 indicate the capability of the three methods in detecting vessels with the challenges shown in Figure 6. As shown in Figure 8(a), for the vessels with stenosis, our algorithm does not overestimate the radius of vessel in stenosis areas. Figure 8(e) shows that our algorithm has the capability of identifying vessel from vessel-like structures, while the other two methods failed to do so. In the regions that contain some parts of vessel-like structures, Schaap's and Cetin's methods go astray, while our algorithm detects the vessel boundary accurately.

### 3.4. Performance Comparison between Cascade-AdaBoost and Cascade-AdaBoost-SVM

We investigate the effect of SVM in the proposed algorithm by comparing the performance of cascade-AdaBoost classifier and cascade-AdaBoost-SVM classifier. The cascade-AdaBoost classifier is obtained by removing SVM classifier from the cascade-AdaBoost-SVM classifier, which is the proposed algorithm. In the training process, we found that the cascade-AdaBoost classifier cannot achieve the targets we set to the cascade-AdaBoost-SVM classifier. Thus, we lower the preset targets for the cascade-AdaBoost classifier as follows: *d* = 0.90, *e* = 0.4, *n*_th_ = 20, and *E*_target_ = *e* − 4.

Table 1 shows the number of weak classifiers on each layer and the layers constructed by the cascade-AdaBoost classifier (CA) and the cascade-AdaBoost-SVM classifier (CAS), respectively. We can observe that the cascade-AdaBoost classifier requests for more weak classifiers on rear layers. Compared with the cascade-AdaBoost-SVM classifier, more layers are needed for the cascade-AdaBoost classifier to achieve the preset target. On some layers in the middle, the cascade-AdaBoost-SVM classifier requires more weak classifiers than the cascade-AdaBoost classifier. That is because the target we set to the cascade-AdaBoost classifier is lower. These observations indicate the cascade-AdaBoost-SVM classifier (our algorithm) is more efficient than the cascade-AdaBoost classifier by adding SVM classifiers.

We also investigate the feature queues selected by these two classifiers on each layer. As shown in Table 2, *f*_1_ and *f*_4_ are selected by the two methods on front layers. This indicates that these two features are more effective in distinguishing tubular structures from nontubular structures than other features. And on rear layers, *f*_2_, *f*_3_, and *f*_5_ are more likely to be used to deal with vessel-like structures, since samples are similar to each other on rear layers.

As we target challenging cases, especially identifying vessels from vessel-like structures, we focus on the feature queues on rear layers (the last two layers). We calculate the utilizations of the vessel features on rear layers for our method as follows: *U*_*f*_1__ = 5.5%, *U*_*f*_2__ = 16.7%, *U*_*f*_3__ = 31.5%, *U*_*f*_4__ = 11.1%, and *U*_*f*_5__ = 35.2%. It can be observed that *f*_3_ and *f*_5_ contribute most in identifying vessels from vessel-like structures as we expect. These two features are described to have the capability of identifying small vessels and reducing noise in the original researches.

**Figure 9:**
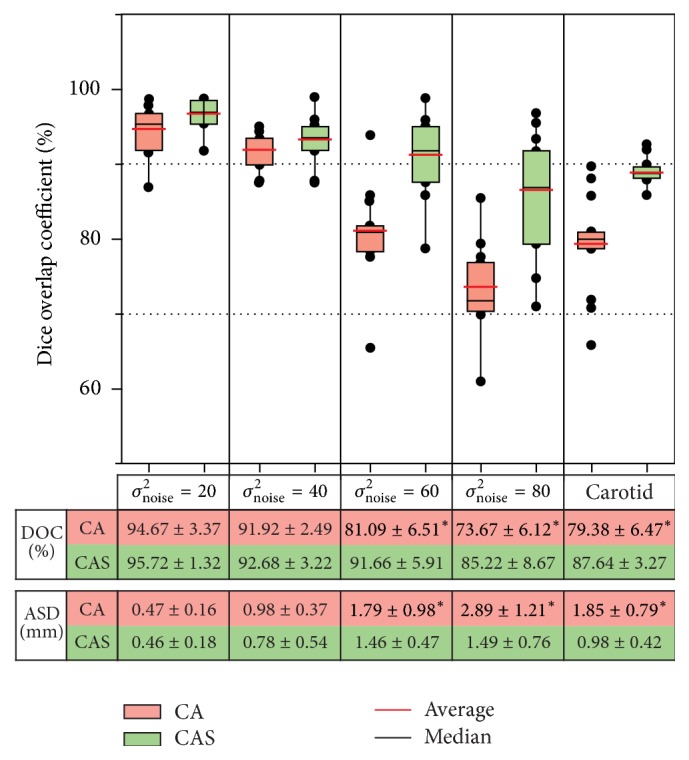
Summary of accuracy evaluation on the synthetic vascular datasets and the carotid artery datasets for the cascade-AdaBoost classifier (CA) and the cascade-AdaBoost-SVM classifier (CAS). DOCs using these two methods are plotted. Average DOC and ASD values and statistical significance are also shown just below the box plot in a table. In the table, symbol *∗* indicates the statistically significant differences between the two methods.

The performance comparison between these two methods is also conducted on the synthetic vascular datasets and the carotid artery datasets, as shown in Figure 9. As the significant differences are indicated by symbol *∗*, we can observe that the addition of SVM improves the segmentation accuracy dramatically in challenging cases (e.g., high levels of noise, blurred boundaries, and vessel-like structures).

## 4. Conclusion

In recent years, many methods [10, 12] have been proposed to segment vessels with the challenges shown in Figure 6. These methods are minimal radius-based or area-based. Facing the presence of stenosis, the shape of the vessel cross-sections is complicated, which makes the minimal radius- or area-based methods inaccurate. They cannot identify vessels with blurred boundaries. Thus, we propose a self-constructing cascade-AdaBoost-SVM classifier for blurred vessel boundaries.

Validation involves comparing published methods, which are with different databases, target different populations, and employ different quality metrics. These differences make the comparison between different methods challenging. Addressing this challenge, we evaluate our method with two state-of-the-art algorithms on the same database. Quantitative analysis for the three algorithms is taken by using the same quality metrics. The comparison results show that our algorithm outperforms the other two algorithms.

In conclusion, we present a self-constructing cascade-AdaBoost-SVM classifier for accurate segmentation of vessel with blurred boundaries and vessel-like structures. This classifier works with a vessel axis + cross-section model, which constrains the classifier around the vessel, and makes the classifier focus on identifying vessels from vessel-like structures. Organizing AdaBoost classifiers and SVM classifiers in a cascade way gives the combined classifier the capability of classifying blurred boundary voxels among similar background voxels. The performance of our method was evaluated on synthetic complex-structured datasets, where we obtained around 91% overlap ratios (DOC). We also validated the proposed method on a challenging application: segmentation of carotid arteries over real patient image datasets. The experimental results with real data are promising, since our method yields better results than two state-of-the-art methods.

## Figures and Tables

**Algorithm 1:**
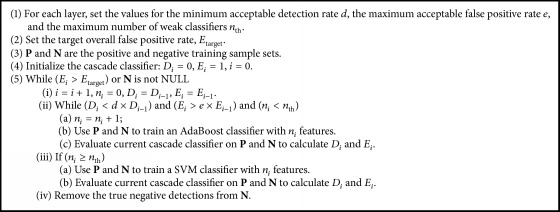
The cascade-AdaBoost-SVM algorithm.

**Table 1:** Weak classifier number on each layer of the cascade-AdaBoost classifier (CA) and the cascade-AdaBoost-SVM classifier (CAS).

Dataset	Classifier	Layer 1	Layer 2	Layer 3	Layer 4	Layer 5	Layer 6	Layer 7	Layer 8	Layer 9	Layer 10
Synthetic	CA	2	2	4	7	14	20	20	20	20	
	CAS	2	2	6	10	SVM	11				

Carotid	CA	2	5	13	20	18	20	20	20	20	19
	CAS	2	8	14	SVM	9	13	SVM			

**Table 2:** The feature queues selected by the cascade-AdaBoost classifier (CA) and the cascade-AdaBoost-SVM classifier (CAS) on each layer.

Dataset	Classifier	Layer 1	Layer 2	Layer 3	Layer 4	Layer 5	Layer 6	Layer 7	⋯
Synthetic	CA	*f*_1_ *f*_4_	*f*_1_ *f*_4_	*f*_4_ *f*_3_ *f*_2_ *f*_3_	*f*_4_ *f*_1_ *f*_3_ *f*_2_ *f*_5_ *f*_2_ *f*_5_	*f*_1_ *f*_3_ *f*_5_ *f*_2_ *f*_4_ *f*_2_ *f*_5_ *f*_3_ *f*_5_ *f*_2_ *f*_4_ *f*_5_ *f*_3_ *f*_2_	*f*_5_ *f*_3_ *f*_2_ *f*_5_ *f*_4_	*f*_4_ *f*_5_ *f*_2_ *f*_5_ *f*_3_	⋯
							*f*_3_ *f*_5_ *f*_2_ *f*_4_ *f*_5_	*f*_2_ *f*_5_ *f*_2_ *f*_1_ *f*_5_	
							*f*_3_ *f*_2_ *f*_3_ *f*_4_ *f*_5_	*f*_2_ *f*_5_ *f*_3_ *f*_2_ *f*_5_	
							*f*_3_ *f*_4_ *f*_5_ *f*_4_ *f*_5_	*f*_2_ *f*_4_ *f*_5_ *f*_3_ *f*_5_	
	CAS	*f*_1_ *f*_4_	*f*_1_ *f*_4_	*f*_1_ *f*_4_ *f*_3_ *f*_2_ *f*_3_ *f*_5_	*f*_4_ *f*_1_ *f*_2_ *f*_5_ *f*_3_ *f*_2_ *f*_3_ *f*_5_ *f*_3_ *f*_5_	*f*_5_ *f*_3_ *f*_5_ *f*_1_ *f*_3_	*f*_5_ *f*_3_ *f*_4_ *f*_5_ *f*_2_		
						*f*_4_ *f*_2_ *f*_3_ *f*_5_ *f*_4_	*f*_3_ *f*_4_ *f*_2_ *f*_3_ *f*_4_		
						*f*_3_ *f*_5_ *f*_3_ *f*_5_ *f*_2_	*f*_5_		

Carotid	CA	*f*_1_ *f*_4_	*f*_4_ *f*_1_ *f*_3_ *f*_5_ *f*_2_	*f*_1_ *f*_3_ *f*_5_ *f*_3_ *f*_5_ *f*_2_ *f*_3_ *f*_1_ *f*_2_ *f*_5_ *f*_2_ *f*_3_ *f*_2_	*f*_4_ *f*_5_ *f*_1_ *f*_5_ *f*_2_	*f*_5_ *f*_3_ *f*_2_ *f*_3_ *f*_5_	*f*_1_ *f*_3_ *f*_2_ *f*_5_ *f*_3_	*f*_1_ *f*_4_ *f*_2_ *f*_3_ *f*_5_	⋯
					*f*_5_ *f*_3_ *f*_5_ *f*_2_ *f*_3_	*f*_3_ *f*_4_ *f*_3_ *f*_5_ *f*_2_	*f*_4_ *f*_2_ *f*_5_ *f*_3_ *f*_2_	*f*_3_ *f*_2_ *f*_3_ *f*_5_ *f*_2_	
					*f*_2_ *f*_5_ *f*_3_ *f*_5_ *f*_3_	*f*_2_ *f*_3_ *f*_2_ *f*_5_ *f*_2_	*f*_3_ *f*_5_ *f*_3_ *f*_5_ *f*_2_	*f*_3_ *f*_2_ *f*_3_ *f*_5_ *f*_3_	
					*f*_1_ *f*_5_ *f*_4_ *f*_5_ *f*_3_	*f*_3_ *f*_2_ *f*_5_	*f*_1_ *f*_5_ *f*_3_ *f*_5_ *f*_3_	*f*_1_ *f*_3_ *f*_4_ *f*_5_ *f*_2_	
	CAS	*f*_1_ *f*_4_	*f*_4_ *f*_1_ *f*_3_ *f*_5_ *f*_3_ *f*_2_ *f*_1_ *f*_5_	*f*_5_ *f*_3_ *f*_4_ *f*_2_ *f*_5_	*f*_4_ *f*_5_ *f*_4_ *f*_5_ *f*_3_	*f*_5_ *f*_4_ *f*_3_ *f*_5_ *f*_2_ *f*_3_ *f*_2_ *f*_3_ *f*_5_	*f*_1_ *f*_5_ *f*_3_ *f*_5_ *f*_2_	*f*_5_ *f*_3_ *f*_2_ *f*_3_ *f*_5_	
				*f*_2_ *f*_3_ *f*_5_ *f*_4_ *f*_5_	*f*_5_ *f*_3_ *f*_5_ *f*_3_ *f*_2_		*f*_3_ *f*_5_ *f*_2_ *f*_3_ *f*_5_	*f*_3_ *f*_1_ *f*_5_ *f*_4_ *f*_5_	
				*f*_1_ *f*_3_ *f*_4_ *f*_5_	*f*_2_ *f*_5_ *f*_3_ *f*_5_ *f*_3_		*f*_3_ *f*_2_ *f*_5_	*f*_3_ *f*_5_ *f*_2_ *f*_5_ *f*_3_	
